# Methanolic extracts of *Withania somnifera* leaves, fruits and roots possess antioxidant properties and antibacterial activities

**DOI:** 10.1186/1472-6882-12-175

**Published:** 2012-10-07

**Authors:** Nadia Alam, Monzur Hossain, Md Abdul Mottalib, Siti Amrah Sulaiman, Siew Hua Gan, Md Ibrahim Khalil

**Affiliations:** 1 Department of Botany, Rajshahi University, Rajshahi, Bangladesh; 2 Laboratory Services Division, Bangladesh Institute of Research and Rehabilitation in Diabetes, Endocrine and Metabolic Disorders Hospital (BIRDEM), Shahbag, Dhaka, Bangladesh; 3 Department of Pharmacology, School of Medical Sciences, Universiti Sains Malaysia, 16150 Kubang Kerian, Kelantan, Malaysia; 4 Human Genome Centre, School of Medical Sciences, Universiti Sains Malaysia, 16150 Kubang Kerian, Kelantan, Malaysia; 5 Department of Biochemistry and Molecular Biology, Jahangirnagar University, Savar, Dhaka, 1342, Bangladesh

**Keywords:** Withania somnifera, Antioxidant, Antibacterial, Free radicals, FRAP

## Abstract

**Background:**

*Withania somnifera,* also known as ashwagandha, is an important herb in ayurvedic and indigenous medical systems. The present study was designed to evaluate the antioxidant and antibacterial activities of an 80% aqueous methanolic extract of *W. somnifera* roots (WSREt), fruits (WSFEt) and leaves (WSLEt).

**Methods:**

Several assays were performed to determine the antioxidant properties of this herb including 1,1-diphenyl-2-picrylhydrazyl radical (DPPH) scavenging activity, ferric reducing antioxidant power (FRAP), ferrous chelation and inhibition of β-carotene bleaching.

**Results:**

The values for DPPH, FRAP, ferrous chelation and inhibition of β carotene bleaching for the three types of extracts ranged from 101.73-801.93 μg/ml, 2.26-3.29 mM Fe/kg, 0.22-0.65 mg/ml and 69.87-79.67%, respectively, indicating that *W. somnifera*, particularly the leaves, possesses significant antioxidant properties. The mean ascorbic acid content was 20.60-62.60 mg/100 g, and the mean anthocyanin content was 2.86-12.50 mg/100 g. Antibacterial activities were measured using the agar well diffusion method and five pathogenic Gram-negative bacteria: *Escherichia coli*, *Salmonella typhi*, *Citrobacter freundii*, *Pseudomonas aeruginosa* and *Klebsiella pneumoniae*. The leaf extracts displayed the highest activity against *S. typhi* (32.00 ± 0.75 mm zone of inhibition), whereas the lowest activity was against *K. pneumoniae* (19.00 ± 1.48 mm zone of inhibition). The lowest minimum inhibitory concentration value was 6.25 mg/ml, which was against *S. typhi*, followed by 12.5 mg/ml against *E. coli*.

**Conclusion:**

In addition to its antioxidant properties, *W. somnifer*a exhibited significant antibacterial activities against Gram-negative bacteria, particularly *S. typhi*.

## Background

Free radical reactions are important factors in the progression of chronic diseases such as cancers, hypertension, cardiac infarction, and atherosclerosis, as well as in rheumatism and cataracts [1]. Many synthetic drugs protect against oxidative damage, but these drugs have adverse side effects [2]. An alternative solution is to consume natural antioxidants from food supplements and traditional medicines [3]. Recently, many natural compounds with antioxidant and antimicrobial properties have been isolated from different plant materials [4].

*W. somnifera* Dunal (*Solanaceae*), also known as ashwagandha or winter cherry, is one of the most valuable plants in the traditional Indian systems of medicine. This plant is used in more than 100 formulations in Ayurveda, Unani and Siddha and is believed to be therapeutically equivalent to ginseng [5]. The ethnopharmacological properties of the plant include adaptogenic, anti-sedative and anti-convulsion activities, and the plant is used to treat various neurological disorders, geriatric debilities, arthritis, stress and behavior-related problems [6].

*W. somnifera* is also used as a dietary supplement because it contains a variety of nutrients and phytochemicals. A decoction of *W. somnifera* roots and leaves is used as a nutrient and health restorative by pregnant women and the elderly. *W. somnifera* thickens and increases the nutritive value of the milk when given to nursing mothers. Additionally, its fruits or seeds are used to curdle plant milk to make vegetarian cheeses [7]. It has been reported that all of the major parts of *W. somnifera* such as the roots, fruits and leaves provide potential benefits for human health because of their high content of polyphenols and antioxidant activities [8]. Previous reports on the activities of methanolic extracts of the whole *W. somnifera* plant against different pathogenic bacteria such as *Escherichia coli, Pseudomonas aeruginosa, Staphylococcus aureus, Streptococcus mutans* and *Candida albicans*, have also found significant antibacterial properties [9].

*W. somnifera* is traditionally used as a therapeutic agent for diarrhea, dyspepsia and gastrointestinal disorders [10]. It has been reported that the antioxidant activities in a plant are dependent on some phytoconstituents such as the phenolic compounds, the anthocyanin and ascorbic acids as well as many other important constituents [11]. In a previous research, the profiles of polyphenols composition in *W. somnifera* roots, fruits and leaves have been reported [8]. In this study, we report the anthocyanin and ascorbic acids contents as well as the antioxidant and antimicrobial properties of *W. somnifera* in different plant parts (the roots, fruits and leaves) to confirm the ethnomedical uses of this medicinal plant as well as to identify the plant part that gives the highest antioxidant activities.

## Methods

### Plant materials

In January 2009, WSR, WSF and WSL were collected from field-grown plants after six months of cultivation in the Botanical Garden of Rajshahi University (Rajshahi, Bangladesh). These plants were identified with the help of the available literature and were authenticated by a botanist, Professor Shah Alam from the Department of Botany, Rajshahi University (Rajshahi, Bangladesh). They were stored at the herbarium lab of the department and the voucher number of the collective plant was WS13. The collected parts of the medicinal plant were cleaned, air-dried in the shade and ground to a fine powder.

### Chemicals and reagents

Reagents such as 1,1-diphenyl-2-picrylhydrazyl radical (DPPH), Folin-Ciocalteu’s reactive, 2,4,6-tris(1-pyridyl)-1,3,5-triazine (TPTZ) and ferrozine [3-(2-pyridyl)-5,6-bis(4-phenylsulfonic acid)-1,2,4-triazine] disodium salt were purchased from Sigma-Aldrich (St. Louis, USA). Sodium acetate trihydrate, glacial acetic acid, hydrochloric acid (HCl), potassium chloride (KCl), iron III chloride ferrous, iron II sulfate, sodium carbonate (Na_2_CO_3_), aluminum chloride (AlCl_3_), ferrous chloride (FeCl_2_), sodium nitrite (NaNO_2_), sodium hydroxide (NaOH), ferric chloride (FeCl_3_.6H_2_O), ferrous sulfate (FeSO_4_⋅7H_2_O), potassium hexacyanoferrate [K_3_Fe(CN)_6_], trichloroacetic acid (TCA), HEPES [4-(2-hydroxyethyl)-1-piperazineethanesulfonic acid] buffer, ethylenediaminetetraacetic acid (EDTA), 2,6-dichlorophenolindophenol, L-ascorbic acid, 3,5-dinitrosalicylic acid (DNSA), β-carotene, linoleic acid, Tween 80 emulsifier and tertiary butylhydroquinone (TBHQ) were purchased from Merck (Darmstadt, Germany). All chemicals used were of analytical grade.

### Preparation of plant extracts

The WSR, WSF and WSL extracts were prepared according to a modified method described by Kahkonen et al. [12]. Ground dry plant materials (500 mg) were weighed in a test tube, followed by the addition of 10 ml of 80% aqueous methanol. The suspension was then gently stirred. The tubes were sonicated for 5 min (45°C) and centrifuged (25°C) for an additional 10 min at 1500 ×*g*. The resulting supernatants were collected. The extraction procedure was repeated three times, and the supernatants were combined before being evaporated using a rotary evaporator to a volume of approximately 1 ml. The concentrated extracts were then lyophilized and weighed. The extracts were resuspended in saline and used as a stock solution.

### Determination of the ascorbic acid content of *W. somnifera*

The ascorbic acid content was determined based on the spectrophotometric method described by Ferreira et al. [13]. Briefly, the sample (100 mg) was extracted with 10 ml of 1% metaphosphoric acid for 45 min at room temperature and filtered through Whatman No. 4 filter paper. The filtrate (1 ml) was then mixed with 9 ml of 2,6-dichlorophenolindophenol, and the absorbance was measured within 30 min at 515 nm against a blank. The content of ascorbic acid was calculated on the basis of the calibration curve of authentic L-ascorbic acid (50, 100, 200, 400 μg/ml; Y = 3.2453X −0.0703; R^2^ = 0.9963), and the results are expressed as mg of ascorbic acid/100 g of *W. somnifera* (dry weight of plant materials).

### Anthocyanin determination

Total anthocyanin content was calculated using the pH differential method [14]. Briefly, aliquots of WSREt, WSFEt and WSLEt (1 ml) were diluted to 10 ml with a pH 1.0 solution (125 ml of 0.2 M KCl and 375 ml of 0.2 M HCl). A second aliquot (1 ml) was diluted to 10 ml with chloride-hydrochloride acid buffered solution (pH 4.5). The absorbance of the solutions was measured at 510 nm, and the concentration (C) of anthocyanins was calculated using the equation below:

(1) $$\text{C mg}/100\;\text{g}=\left[\left(\text{Abs pH}\;1.0–\text{Abs pH}\;4.5\right)\times 484.82\times 1000/24825\right]\times \text{DF}$$

Where the term in parentheses represents the difference between the absorbance (Abs) at 510 nm of the pH 1.0 and pH 4.5 solutions, 484.82 is the molecular mass of cyanidin-3-glucoside chloride, 24,825 is the molar absorptivity (ε) nm in the pH 1.0 solution, and DF is the dilution factor. Each experiment was performed in duplicate. The results are expressed as mg of anthocyanin/100 g of *W. somnifera* (dry weight of plant materials).

### DPPH free radical scavenging activity

The antioxidant capacities of the *W. somnifera* extracts were also studied by evaluating their free radical scavenging effects on the DPPH radical, which was based on the method proposed by Ferreira et al. [15]. Briefly, 1 ml of WSREt, WSFEt or WSLEt (1 mg/ml each) was mixed with 2.7 ml of a methanol solution containing DPPH radicals (0.024 mg/ml). The mixture was vigorously shaken and incubated at room temperature in the dark for 60 min, at which time their absorbances remained unchanged. Reduction of the DPPH radical was determined by measuring the absorbance at 517 nm [16]. Radical scavenging activity (RSA) was calculated as the percentage of DPPH discoloration using the following equation: *%* RSA=[(A_DPPH_−A_S_)/A_DPPH_] × 100, where A_S_ is the absorbance of the solution when the sample extract has been added at a particular level and A_DPPH_ is the absorbance of the DPPH solution. The 50% maximal inhibitory concentration (IC_50_) was determined as the concentration of the tested WSREt, WSFEt or WSLEt sample resulting in a 50% reduction of the initial DPPH concentration, measured from the linear regression concentration curve of the test extract (μg/ml) against the percentage of the radical scavenging inhibition.

### Ferric reducing/antioxidant power assay (FRAP assay)

The FRAP assay was performed according to a modified method described by Benzie and Strain [17]. Briefly, 200 μL of WSREt, WSFEt or WSLEt (1 mg/ml) was mixed with 1.5 ml of the FRAP reagent, and the reaction mixture was incubated at 37°C for 4 min. The absorbance was then read at 593 nm against a blank that was prepared using distilled water. The preparation of FRAP reagents was as follows: (i) Reagent A (300 mM/l acetate buffer (pH 3.6)); (ii) Reagent B, TPTZ solution, (0.031 g of TPTZ was added to 10 ml of 40 mM HCl and dissolved at 50°C); and (iii) Reagent C, ferric chloride solution, 0.54 g of ferric chloride was dissolved in 10 ml of distilled water. To make the FRAP reagent (30 ml), 2.5 ml of reagent B and 2.5 ml of reagent C were added to 25 ml of reagent A. The FRAP reagent was pre-warmed to 37°C. Reagents B and C were freshly prepared immediately before the assay was performed. A calibration curve was generated using aqueous solutions of FeSO_4_ at 100, 200, 400, 600 and 1000 μM. Ascorbic acid and BHT were used as positive controls. FRAP values were expressed as micromoles of ferrous equivalent [mmole Fe (II)] per kg of *W. somnifera* extract (DW).

### Ferrous ion (Fe^2+^) chelation

Ferrous ion chelating activity was evaluated using a standard method [18] with minor modifications. The reaction was performed in HEPES buffer (20 mM, pH 7.2). Briefly, different concentrations (0, 7.5, 15, 30, 60, or 120 μg/ml) of plant extract were added to 12.5 μM ferrous sulfate solution, and the reaction was initiated by the addition of ferrozine (75 μM). Ferrozine was dissolved in water to a concentration of 5 mg/ml. The mixture was shaken vigorously and incubated for 20 min at room temperature. The absorbance was then measured at 562 nm [19]. All tests were performed in triplicate, and mean values are reported. EDTA was used as a positive control. The percentage of inhibition of ferrozine–Fe^2+^ complex formation was calculated using the following formula:

(2) $${\text{Bound Fe}}^{2+}\left(\%\right)=\left[1-\left(\lambda_{562}-\text{S}\right)\right]/\left(\lambda_{562}-\text{C}\right)\;\text{X}\;100$$

Where λ_562_-C is the absorbance of the control and λ_562_-S is the absorbance in the presence of WSREt, WSFEt, WSLEt or the standards. The control contained only FeCl_2_ and ferrozine [20]. The 50% maximal inhibitory concentration (IC_50_) was determined as the concentration of the tested WSREt, WSFEt or WSLEt sample causing 50% inhibition of ferrozine–Fe^2+^ complex formation, measured from the linear regression concentration curve of the test extract (mg/ml) against the percentage of bound Fe^2+^.

### Inhibition of β-carotene bleaching

The antioxidant activities of the methanolic extracts of *W. somnifera* were evaluated using the β-carotene linoleate model system. A solution of β-carotene was prepared by dissolving 2 mg of β-carotene in 10 ml of chloroform. Two milliliters of this solution was pipetted into a 100 ml round-bottom flask. After the chloroform was removed at 40°C under vacuum, 45 μl of linoleic acid, 400 μl of Tween 80 emulsifier and 100 ml of distilled water were added to the flask with vigorous shaking. Aliquots (4.8 ml) of this emulsion were transferred into different test tubes containing 0.2 ml of various concentrations of the *W. somnifera* solutions or phenolic extracts. The tubes were shaken and incubated at 50°C in a water bath. Upon the addition of the emulsion to each tube, the time zero absorbance was measured at 470 nm using a spectrophotometer. The absorbance was then recorded at 20 min intervals until the control sample changed color. A blank, devoid of β-carotene, was prepared for background subtraction [21].

Lipid peroxidation (LPO) inhibition was calculated using the following equation:

*%* of LPO inhibition = (*β* − carotene content; after 2 hr of assay/initial *β* − carotene content) × 100. Ascorbic acid and TBHQ were used as positive controls.

### Antibacterial properties of *W. somnifera*

#### Extract sterility

The extracts were filtered using Millipore nylon membranes (0.45 μm) and then tested for sterility by introducing 2 ml of the extract into 10 ml of sterile nutrient broth. The extracts were incubated at 37°C for 24 hr. A sterile extract was indicated by the absence of turbidity or the clarity of the broth after the incubation period [22].

#### Bacterial strains

The *in vitro* antimicrobial activities of WSREt, WSFEt and WSLEt were investigated. Five pathogenic bacteria, namely *Escherichia coli*, *Salmonella typhi*, *Citrobacter freundii*, *Pseudomonas aeruginosa* and *Klebsiella pneumoniae,* were obtained from the Bangladesh Institute for Research and Rehabilitation in Diabetes, Endocrine and Metabolic Disorders (BIRDEM). All of the microorganisms were maintained at 4°C on nutrient agar slants. The bacterial strains were re-identified on the basis of their morphological, cultural and biochemical characteristics [23] as an additional confirmatory step.

#### Determination of antibacterial activity

The antibacterial activities of WSREt, WSFEt and WSLEt were determined using the agar well diffusion method following a published procedure with slight modifications [24]. Briefly, for the evaluation of antimicrobial activities, a fresh 24 hr culture of bacteria was suspended in sterile distilled water to obtain a turbidity of 0.5 McFarland units. The final inoculum size was adjusted to 5×10^5^ CFU/ml. Nutrient agar (nutrient broth+1.8% agar) was inoculated with the given microorganism by spreading the bacterial inoculum on the media. Wells (8 mm diameter) were punched in the agar and filled with 200 μl of the plant extracts (5 mg/ml). Negative control wells containing neat solvent (80% aqueous methanol) or a standard antibiotic solution of tetracycline (100 μg/ml) (positive control) were run in parallel on the same plate. The plates were incubated at 37°C for 24 hr. Antibacterial activities were assessed by measuring the diameters of the zones of inhibition for the respective drugs. The relative antibacterial potency of a given preparation was calculated by comparing its zone of inhibition with that of the standard antibiotic tetracycline.

#### Determination of the minimum inhibitory concentration (MIC) of the extract

The initial plant extract (100 mg/ml) was serially diluted by transferring 5 ml of the sterile plant extract (stock solution) into 5 ml of sterile nutrient broth to obtain the following dilutions: : 25 mg/ml, 12.5 mg/ml, 6.25 mg/ml and finally 3.125 mg/ml [25]. After obtaining the different extract concentrations, each concentration was inoculated with 0.1 ml of a standardized bacterial cell suspension (approximately 10^6^ CFU/ml) and incubated at 37°C for 24 hr. The lowest concentration of the extract that inhibited the growth of the test organism was taken as the MIC. The controls were prepared as follows: (i) nutrient broth only (positive control), (ii) nutrient broth and sterile plant extract, (iii) nutrient broth and a test organism (positive control), and (iv) the standard antibiotic tetracycline (positive control).

#### Statistical analyses

All analyses were performed in triplicate, and the data are expressed as the mean ± standard deviation (SD). The data were analyzed using the Statistical Package for the Social Sciences Version 12.0 (SPSS Inc., USA) and MS Excel 2003. One-way analysis of variance (ANOVA) followed by Tukey’s honestly significant difference post hoc test was used to compare the data. Differences of *P* < 0.05 were considered significant.

## Results

### Ascorbic acid contents of WSREt, WSFEt and WSLEt

The highest concentration of ascorbic acid was found in WSLEt. WSREt had the lowest ascorbic acid concentration, and an intermediate concentration of ascorbic acid was found in WSFEt (Table 1).

**Table 1:** Ascorbic acid and anthocyanin contents (mg/ 100 g dry weight plant materials) in WSREt, WSFEt and WSLEt

**Sample**	**Ascorbic acid**	**Anthocyanin**
WSLEt	62.60±1.26^a^	12.56±1.04^a^
WSREt	20.60±1.06^c^	2.86±1.44^c^
WSFEt	40.00±0.40^b^	5.66±0.52^b^

Different letters in each column indicate a significant difference (p<0.05).

### Anthocyanin contents

The highest concentration of anthocyanins was also found in WSLEt (12.5 ± 1.04 mg/ 100 g), and WSREt had the lowest concentration (2.86 ± 1.44 mg/ 100 g) (Table 1).

### DPPH radical scavenging activity (IC_50_)

The DPPH stable free radical method is an easy, rapid and sensitive way to survey the antioxidant activity of a specific compound or plant extract [26]. The IC_50_ values for the DPPH radical scavenging activities of WSLEt, WSREt and WSFEt are shown in Table 2 in comparison with the ascorbic acid and BHT controls.

**Table 2:** **Results of DPPH radical scavenging activity (IC**_**50**_**), FRAP assay, Ferrous chelation activity and Inhibition of lipid peroxidation (LPO)**

**Sample**	**DPPH IC**_**50**_**(μg/ml)**	**FRAP (mmole Fe/kg)**	**Fe**^**2+**^**chelation IC**_**50**_**(mg/ml)**	**% of LPO inhibition**
WSLEt	101.73±8.96^c^	3.29±8.03^c^	0.22±0.04^c^	79.67±3.85^c^
WSREt	801.93±7.92^a^	2.26±1.46^e^	0.37±0.02^b^	69.87±4.85^d^
WSFEt	345.68±8.98^b^	3.13±21.52^d^	0.65±0.02^a^	72.11±6.40^d^
Ascorbic acid	25.95±1.08^d^	10.86±9.52^b^	NA	100.28±1.07^b^
BHT	38.14±0.89^d^	12.57±13.23^a^	NA	NA
EDTA	NA	NA	0.13±0.01^d^	NA
TBHQ	NA	NA	NA	104.516±6.34^a^

Different letters in each column indicate a significant difference (p<0.05). NA = Not applicable.

### FRAP assay

The FRAP assay provides a direct estimation of the level of antioxidants or reductants present in a sample and is based on the ability of the analyte to reduce the Fe^3+^/Fe^2+^ pair. WSLEt exhibited the highest FRAP value (3.29 mM Fe/kg), whereas WSREt had the lowest (2.26 μM Fe/kg). Ascorbic acid and BHT were used as positive controls, and the FRAP values of these controls were higher when compared to all three plant parts (Table 2). These results indicate that the leaves are a good potential source of antioxidants, followed by the fruits and roots.

### Ferrous chelation activity

As an additional approach, the ferrous chelation assay was performed. Ferrozine produces a violet complex with Fe^2+^, and in the presence of a chelating agent, complex formation is interrupted, resulting in a decrease of the intensity of the violet color. The results demonstrate that formation of the ferrozine-Fe^2+^ complex was inhibited in the presence of the test and reference compounds (Table 2).

### Inhibition of β-carotene bleaching

The effect of 60 μg/ml of WSLEt, WSREt, WSFEt and the reference compounds ascorbic acid and TBHQ on the lipid peroxidation of a linoleic acid emulsion is shown in Table 2. Peroxidation of the linoleic acid emulsion was inhibited by 79.67, 69.87 and 72.11% when WSLEt, WSREt and WSFEt, respectively, were used, indicating that the leaves have the highest antioxidant properties. The reference compounds ascorbic acid and TBHQ exhibited 100.28% and 104.52% inhibition of peroxidation, respectively.

### Antimicrobial properties

As shown in Table 3, the antimicrobial activities of WSREt, WSFEt and WSLEt and their potencies were quantitatively assessed by the presence or absence of a zone of inhibition and the zone diameter, respectively. The methanolic extracts (80%) of the three plant parts displayed antimicrobial activities against all five pathogenic bacteria. WSLEt displayed the highest antibacterial activity against all of the pathogenic bacteria tested compared with WSREt and WSFEt.

**Table 3:** Diameters of the zones of inhibition for the 80% aqueous methanolic extracts of WSREt, WSFEt and WSLEt on several species of Gram-negative bacteria

**Test organisms**	**Diameters of zones of inhibition (mm)**			
	**WSREt**	**WSFEt**	**WSLEt**	**Tetracycline (100 μg/ml)**
*Escherichia coli*	15.00 ± 0.96^c^	12.00 ± 1.50^d^	28.00 ± 0.56^b^	43.00 ± 1.90^a^
*Salmonella typhi*	13.00 ± 0.43^c^	14.00 ± 0.79^c^	32.00 ± 0.75^b^	47.00 ± 1.42 ^a^
*Citrobacter freundii*	11.00 ± 0.86^c^	9.00 ± 1.21^c^	23.00 ± 1.27^b^	38.00 ± 1.22 ^a^
*Pseudomonas aeruginosa*	9.00 ± 1.32^c^	10.00 ± 1.43^c^	26.00 ± 1.08^b^	40.00 ± 0.59 ^a^
*Klebsiella pneumoniae*	10.00 ± 1.10^c^	8.00 ± 1.61^c^	19.00 ± 1.48^b^	36.00 ± 1.13 ^a^

Each value represents the mean ± SD of three different observations. Different letters in each row indicate a significant difference (p<0.05).

Overall, WSLEt exhibited the greatest zone of inhibition against all five microorganisms. For the specific plant parts, WSLEt exhibited the highest activity against *S. typhi*, whereas the lowest activity was against *K. pneumoniae*. The largest zone of inhibition for WSFEt was against *S. typhi*, and the smallest zone of inhibition was also against *K. pneumoniae*. For WSREt, the largest zone of inhibition was against *E. coli*, whereas the smallest zone of inhibition was against *P. aeruginosa.*

The MICs for WSREt, WSFEt and WSLEt against the five pathogenic bacteria are shown in Table 4. WSLEt had the lowest MIC against *S. typhi*, whereas its highest MIC values were against *C. freundii, P. aeruginosa* and *K. pneumonia*. The lowest MIC for WSFEt (25.00 mg/ml) was found for *E. coli* and *S. typhi*, whereas MICs of 50 mg/ml were found for *C. freundii, P. aeruginosa* and *K. pneumoniae*. For WSREt, the lowest MIC (25.00 mg/ml) was found for *E. coli,* whereas its MIC was similar for the four other bacterial species, that is, 50.00 mg/ml.

**Table 4:** Determination of the minimum inhibitory concentration (MIC) of the methanolic extracts of the, WSFEt and WSLEt on the test organisms

**Test organisms**	**Concentrations (mg/ml)**			
	**WSREt**	**WSFEt**	**WSLEt**	**Tetracycline**
*Escherichia coli*	25.00	25.00	12.50	0.03
*Salmonella typhi*	50.00	25.00	6.25	0.01
*Citrobacter freundii*	50.00	50.00	25.00	0.05
*Pseudomonas aeruginosa*	50.00	50.00	25.00	0.05
*Klebsiella pneumoniae*	50.00	50.00	25.00	0.03

## Discussion

This study is the first to report and compare the antioxidant and antibacterial properties of the different parts of *W. somnifera*, namely the leaves, roots and fruits*.* Our study is also the first to report on the ascorbic acid levels of WSFEt and WSLEt as well as the anthocyanin concentrations present in *W. somnifera.* We found that *W. somnifera* leaves have the highest antioxidant and antimicrobial activities as well as the highest ascorbic acid and anthocyanin contents. These observations may be related to the high phenolic and flavonoid contents reported in our previous study [8,27].

The highest concentration of ascorbic acid was found in WSLEt, whereas WSREt had the lowest ascorbic acid content. Hussain et al., [28] reported a higher vitamin C content (51.50 mg/ 100 g) in WSREt. In another study [29], the ascorbic acid contents of the leaves and roots of *W. somnifera* were compared, and the roots were found to possess a slightly higher ascorbic acid content. This variability may be due to differences in the source of the *W. somnifera* plant used. Anthocyanins are flavonoids [30] and are among the important phenolic substances in *W. somnifera*. Flavonoids are polyphenolic compounds with known properties that include free radical scavenging, inhibition of hydrolytic and oxidative enzymes and anti-inflammatory action [31]. The mechanisms of action of flavonoids are through scavenging or chelating processes [32]. It has been reported that the anthocyanins present in *W. somnifera* are chemically-similar to the ones present in black raspberry (*Rubus occidentalis*) [33]. As with ascorbic acid, the content of total anthocyanins in WSLEt, WSREt and WSFEt varies in different parts of the plant and is highest in the leaves. However, this level is lower than the highest concentration of anthocyanins found in tomato, which is 2.83 ± 0.46 mg/g [34].

Free radical DPPH scavenging ability is extensively used to indicate the antioxidant potential of naturally derived foods and plants. We found that *W. somnifera* exhibited good DPPH free radical scavenging abilities. WSLEt had the lowest IC_50_. IC_50_ values between 206.77 and 224.96 μg/ml [35] have been reported in previous studies involving only *W. somnifera* leaves. These researchers indicated that the IC_50_ values for plant extracts harvested from polluted sites are higher when compared with those harvested from non-polluted sites.

A relatively higher FRAP value, as was determined for WSLEt, indicated a greater reduction of ferric ions to ferrous ions and higher antioxidant properties in the leaves when compared with the other plant parts (Table 2).

Metal chelating capacity is important because it reduces the concentration of transition metals that catalyze lipid peroxidation [36]. Ferrous chelation tests were performed, and the results showed that although WSREt, WSFEt and WSLEt did not display chelation activities as high as that of the EDTA standard (Table 2), the decrease in the concentration-dependent color formation in the presence of the extracts indicated that they did exhibit some amount of iron-chelating activity.

However, *W. somnifera* extracts exhibited high inhibitory effects to lipid peroxidation of linoleic acid. The activities of antioxidants have been attributed to various mechanisms such as prevention of chain initiation, decomposition of peroxides, reducing capacity and radical scavenging [37]. High inhibitory effect of lipid peroxidation of the extracts could be due to the abundant presence of antioxidant active compounds. Therefore, these extracts may be effective agents in retarding Fe^2+^ catalyzed lipid oxidation.

These results indicate that the antioxidant capacity of each extract may be related to the concentrations of ascorbic acid, anthocyanin and polyphenols. The antioxidant activities of these compounds depends on their molecular structures, that is, on the availability of phenolic hydrogens which result in the formation of phenoxyl radicals due to hydrogen donation [38].

For the antibacterial activity study, the 80% methanolic extract of all parts of *W. somnifera* displayed activity against five pathogenic Gram-negative bacteria, namely *E. coli*, *S. typhi, C. freundii, P. aeruginosa* and *K. pneumoniae*, to different magnitudes. *W. somnifera* leaves possessed the greatest antimicrobial effects*.* Phenolics, ascorbic acid and anthocyanins are associated with the antimicrobial efficiency of the plant because they cause hyperacidification at the plasma membrane interface of the pathogen, which potentially results in the disruption of the H^+^-ATPase required for ATP synthesis [39].

The most susceptible organism was *S. typhi*, indicating that the *W. somnifera* extracts contain active compounds that can inhibit the proliferation and growth of *S. typhi.* which can cause diseases such as typhoid fever and foodborne illnesses. This finding may support the traditional uses of *W. somnifera* as a therapeutic agent for diarrhea, dyspepsia and gastrointestinal disorders [10]. The other tested bacteria also exhibited significant sensitivities against WSREt, WSFEt and WSLEt. These results demonstrate that the methanolic extracts contained the expected compounds for antibacterial activities against the five tested Gram-negative bacteria. *W. somnifera* may be exploited as a natural drug for the treatment of several infectious diseases initiated by these organisms. This finding is important in the quest for new antimicrobial agents because organisms with multidrug resistance are rapidly emerging.

Jain and Varshney [9] reported on the antibacterial activity of the methanolic extracts of the whole *W. somnifera* plant against *Escherichia coli, Pseudomonas aeruginosa, Staphylococcus aureus, Streptococcus mutans* and *Candida albicans*, with zones of inhibition of 38, 36, 15, 38 and 32 mm, respectively. These results are similar to our reported zones of inhibition for WSLEt against two of the same organisms, *Escherichia coli* and *Pseudomonas aeruginosa,* at 28 ± 0.56 and 26 ± 1.08 mm, respectively. However, Jain and Varshney [9] reported that aqueous extracts of *W. somnifera* had higher antimicrobial activities (a zone of inhibition between 33 and 50 mm) when compared with methanolic extracts. In this study, methanol was used to extract low molecular weight and moderately polar substances because of its wide range of solubility. However, Jain and Varshney [9] did not report any MIC values for comparison with our own values.

Ascorbic acid, anthocyanin and polyphenols have been reported to exhibit antibacterial activities with distinguishing characteristics for their reactivities with proteins related to polyamide polymers [40]. The inhibition of microorganisms by these antioxidant compounds may be due to iron deprivation or hydrogen bonding with vital proteins such as microbial enzymes [41]. Antioxidant compounds such as ascorbic acid, anthocyanin and polyphenols are vulnerable to polymerization in air through oxidization reactions. Therefore, an important factor governing their toxicity is their polymerization size. It has been reported that the oxidized condensation of antioxidant compounds may result in the toxification of microorganisms [41]. These findings support the fact that ascorbic acid, anthocyanin and polyphenols may be responsible for the antimicrobial activities of WSREt, WSFEt and WSLEt.

The phytochemical constituents quantified in the present study such as ascorbic acid as well as anthocyanin and as also determined in our previous study i.e. the polyphenols and flavonoids [8] are of significant medicinal importance and may act as antioxidant, antimicrobial and immunomodulatory agents. In the future, the above phytoconstituents could be used as a major tool for obtaining a quality control profile for a drug.

## Conclusion

Our results clearly indicate that *W. somnifera*, particularly the leaves, has remarkable antioxidant properties. Additionally, the leaves possess significant antibacterial properties against Gram-negative organisms, in particular, *S. typhi*. It will be beneficial to investigate the active compounds present in *W. somnifera* so that its leaves can be used to increase the armamentarium of antimicrobial agents and so that other possible therapeutic uses of the plant can be explored.

## Competing interests

The authors declare no competing interests.

## Authors’ contributions

NA, MIK and MAM performed the experiments. MH, SAS and GSH supervised the work, evaluated the results and revised the manuscript for publication. All the authors read and approved the final manuscript.

## Pre-publication history

The pre-publication history for this paper can be accessed here:

http://www.biomedcentral.com/1472-6882/12/175/prepub
